# Severe *NDE1*-mediated microcephaly results from neural progenitor cell cycle arrests at multiple specific stages

**DOI:** 10.1038/ncomms12551

**Published:** 2016-08-24

**Authors:** David J. Doobin, Shahrnaz Kemal, Tiago J. Dantas, Richard B. Vallee

**Affiliations:** 1 Department of Pathology and Cell Biology, Columbia University, New York, New York 10032, USA

## Abstract

Microcephaly is a cortical malformation disorder characterized by an abnormally small brain. Recent studies have revealed severe cases of microcephaly resulting from human mutations in the *NDE1* gene, which is involved in the regulation of cytoplasmic dynein. Here using *in utero* electroporation of NDE1 short hairpin RNA (shRNA) in embryonic rat brains, we observe cell cycle arrest of proliferating neural progenitors at three distinct stages: during apical interkinetic nuclear migration, at the G2-to-M transition and in regulation of primary cilia at the G1-to-S transition. RNAi against the NDE1 paralogue NDEL1 has no such effects. However, NDEL1 overexpression can functionally compensate for NDE1, except at the G2-to-M transition, revealing a unique NDE1 role. In contrast, NDE1 and NDEL1 RNAi have comparable effects on postmitotic neuronal migration. These results reveal that the severity of *NDE1*-associated microcephaly results not from defects in mitosis, but rather the inability of neural progenitors to ever reach this stage.

## Abstract (web summary)

Human mutations in the *NDE1* gene have been associated with cortical malformations and severe microcephaly. Here, the authors show in embryonic rat brains that *NDE1*-depleted neural progenitors arrest at three specific cell cycle stages before mitosis, resulting in a severe decrease in neurogenesis.

## Graphical abstract

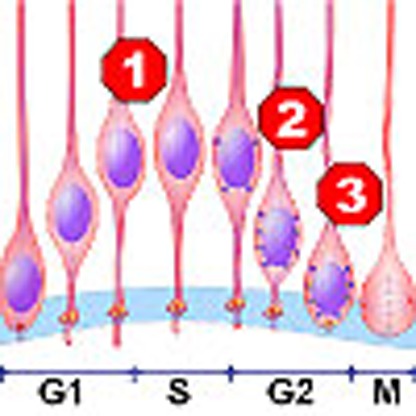

Autosomal-recessive primary microcephaly is a severe developmental condition characterized by small brain size and a substantial reduction in neuronal number^1^, with the cerebral cortex most prominently affected. Laminar organization is often normal, though microcephaly can occasionally be accompanied by lissencephaly or other organization defects^1^,^2^. A number of microcephaly genes have been identified, including *ASPM*, *microcephalin*/*BRIT1*, *CDK5RAP2*/*Cep215*, *CENPJ*/*CPAP*, *SIL*/*STIL*, *WDR62*, and *NDE1* (refs 3, 4, 5). Several of these genes are associated with centrosome and/or mitotic function, suggesting that errors in neural progenitor cell proliferation contribute to disease pathology.

*NDE1* was an early candidate gene for microcephaly as judged from mouse studies^6^, and has subsequently been implicated in a particularly severe form of microcephaly and microlissencephaly in human patients^2^,^7^,^8^,^9^,^10^. NDE1 and its paralogue, NDEL1, exhibit clear homology to *Aspergillus* NudE (Nuclear Distribution E)^11^, and function along with LIS1 in cytoplasmic dynein regulation^12^,^13^,^14^,^15^. The *NDE1* null mouse was reported to exhibit ectopic mitotic divisions accompanied by altered mitotic spindle orientation^6^. Roles for NDE1 and NDEL1 in mitosis have been borne out by analysis of non-neuronal cells *in vitro*^16^,^17^,^18^,^19^. However, human *NDE1*-associated microcephaly is much more severe than the forms of the disorder involving mitotic spindle assembly genes, such as *ASPM* and *WDR62* (refs 4, 5). This observation suggests that NDE1 might be involved in more than one aspect of neural progenitor proliferation. In addition, patients with *NDE1* mutations often exhibit microcephaly with lissencephalic features^2^,^7^, suggesting potential roles for NDE1 during subsequent neuronal migration as well.

Mammalian neocortical development begins with the expansion of neuroepithelial cells within the neural tube, followed by formation of the layered neocortex^20^,^21^. The apical-most region, which is adjacent to lateral ventricle and defined as the ventricular zone (VZ), is populated by the soma of radial glia progenitor (RGP) cells^22^. These serve as stem cells, responsible for the production of all excitatory cortical neurons, most glial cells and adult stem cells^20^,^23^. The RGP cells are highly elongated, with their apical and basal processes spanning the entire thickness of the developing neocortex. A hallmark of RGP cell behaviour is the cell cycle-linked oscillatory movement of the nucleus of RGP cells, termed interkinetic nuclear migration (INM)^24^. RGP mitosis occurs exclusively at the ventricle^25^. The RGP nucleus then migrates basally during G1, progresses through S-phase in the upper portion of the VZ and then migrates apically during G2 toward the ventricle where the next mitotic division occurs^26^. Mitosis can be symmetric, resulting in self-renewal of the neural progenitor pool, or asymmetric, leading to one neural progenitor and either a post-mitotic neuron or an intermediate progenitor, each of which migrate away from the ventricle.

Our own studies revealed that knockdown of genes involved in apical INM prevent RGP nuclei from reaching the ventricle and undergoing mitosis^25^,^27^,^28^,^29^,^30^,^31^,^32^. We found that apical migration is mediated by cytoplasmic dynein anchored to the nuclear envelope during G2, which carries the nucleus along a polarized microtubule network emanating from the apically anchored centrosome^25^,^29^. Dynein is recruited to the nuclear envelope by two G2-specific nuclear pore-mediated mechanisms in a Cdk1-dependent manner^31^. The first mechanism is activated during early G2 and involves BicD2 binding to the nucleoporin RanBP2 (ref. 33), whereas the second mechanism is activated during late G2 and depends on CENP-F binding the nucleoporin Nup133 (refs 31, 34). On the basis of the restriction of nuclear-envelope NDE1/NDEL1 signal to late-G2 in HeLa cells^31^, we envision that either NDE1, NDEL1, or both, might contribute to late apical nuclear migration in the developing brain, though this possibility has not been tested.

Both NDE1 and NDEL1 mRNAs and protein have been detected throughout the developing neocortex (Allen Developing Mouse Brain Atlas, http://developingmouse.brain-map.org/)^13^,^35^. However, NDE1 is more highly expressed in areas of high proliferation, such as the VZ, whereas the highest levels of NDEL1 mRNA were detected in the cortical plate (CP), where neuronal migration takes place (Allen Developing Mouse Brain Atlas, http://developingmouse.brain-map.org/). NDEL1 has not been implicated in microcephaly, but rather in later aspects of neuronal migration in animal models^13^,^14^. These observations are consistent either with the different expression patterns for NDE1 and NDEL1 in the developing brain, or with differences in their function. Some evidence for NDEL1-specific roles has been reported in cultured cells^36^, but the relative contributions of the two genes to neocortical development remains to be explored^37^.

The current study was initiated to determine which NDE1 roles contribute most significantly to the extreme form of microcephaly seen in human patients. In addition, we investigated potential roles for NDEL1 during neocortical development, and tested the degree to which these two proteins can functionally compensate for one another. Through use of RNA interference (RNAi) against each paralogue, we find that both NDE1 and NDEL1 contribute to postmitotic neuronal migration. However, NDE1 inhibition alone causes a complete block of apical INM, contributing to a large reduction in mitotic index with no evidence for ectopic mitoses. We also find a potent block of the G1-to-S transition associated with primary cilia over-elongation after the co-depletion of NDE1 and NDEL1. Interestingly, NDEL1 expression rescues most, but not all, of NDE1 functions, identifying a NDE1-specific role, after INM completion at a novel premitotic state. These results identify multiple new potential causes for microcephaly, and provide a basis for understanding the distinct pathogenic potential of NDE1 versus NDEL1.

## Results

### Common and distinct effects of NDE1 and NDEL1 RNAi

**Figure 1:**
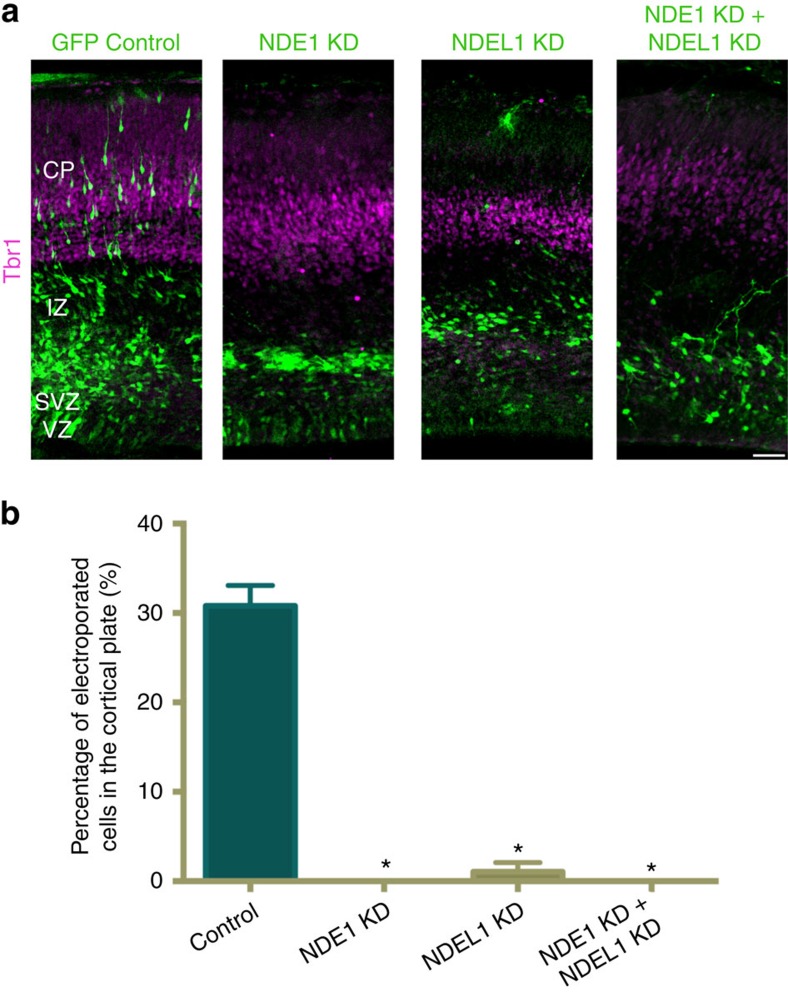
Effects of NDE1 and NDEL1 RNAi on neuronal migration into the cortical plate. (**a**) Representative images of embryonic day 20 (E20) rat neocortex with a control vector expressing GFP alone, or shRNAs to NDE1, NDEL1, or both genes along with a GFP reporter. Sections were stained for Tbr1 to mark neurons in the cortical plate (CP). Scale bar, 50 μm. (**b**) Quantification of the fraction of electroporated cells in the CP across NDE1, NDEL1 or combined RNAi conditions 4 days post electroporation at E16. All knockdown conditions nearly eliminated any cells from reaching the CP, in comparison with control neurons. Data are presented as mean±s.e.m., unpaired t-tests used for all comparisons. *P<0.05, *n*=3 embryonic brains from different mothers.

To address the specific roles of NDE1 or NDEL1 during neocortical development, we used *in utero* electroporation of embryonic day 16 (E16) rat brain to introduce plasmids expressing GFP alone or in combination with NDE1 or NDEL1 shRNAs. Reduction of protein levels (Supplementary Fig. 1A and Supplementary Fig. 8) and mRNA levels (Supplementary Fig. 1B) were confirmed in rat C6 glioma cells. Relative to control, NDE1 knockdown caused a nearly complete loss of transfected neurons from reaching the CP, which instead accumulated in the lower intermediate zone (IZ) and subventricular zone (SVZ; Fig. 1a,b). NDEL1 knockdown also prevented cells from reaching the CP, though some cells were observed in the upper IZ, and there was again a large relative accumulation in the lower IZ and SVZ (Fig. 1a,b). The effects of dual NDE1/NDEL1 knockdown were similar to those for NDE1 and NDEL1 alone (Fig. 1a,b).

**Figure 2:**
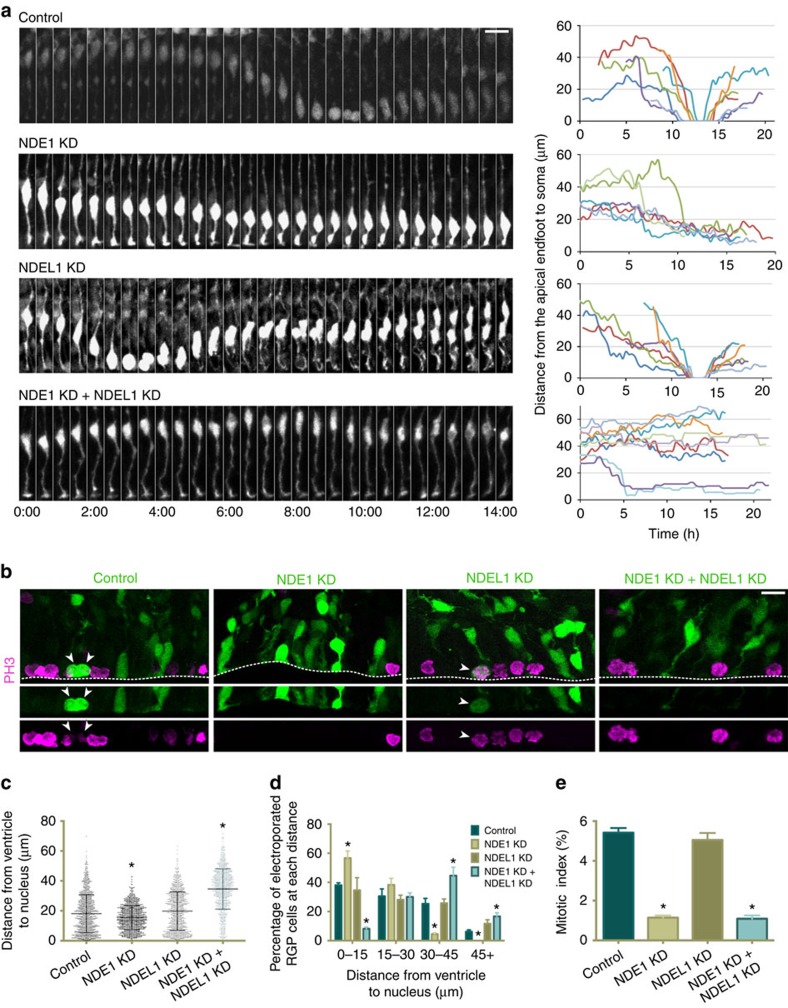
NDE1 knockdown blocks apical nuclear migration and potently reduces the mitotic index. (**a**) Live-imaging montage of GFP-expressing RGP cells at E19 with a control empty vector expressing GFP alone, or shRNAs to NDE1, NDEL1 or both genes along with a GFP reporter. Representative tracings from multiple RGP cells for each condition are shown at right. Montage panels are shown at 30 min intervals (Supplementary Movies 1–4). (**b**) Representative images of the VZ from the electroporated brains stained for the mitotic marker phosphohistone-H3 (PH3). Arrowheads mark soma of PH3+/GFP+ RGP cells. Dashed line represents the ventricular surface. (**c**,**d**) Measurements of the distance between the bottom of the nucleus and the ventricular surface, corresponding to the apical process length, across the various conditions. NDE1 knockdown shifted the apical process length distribution towards shorter distances, with a significant accumulation of RGPs with an apical process of 0–15 μm. NDE1/NDEL1 double knockdown, however, shifted the apical process length distribution to larger distances, with a significant accumulation of RGPs with an apical process of 30–45 μm. Each dot represents an individual apical process length measurement for one electroporated RGP cell. (**e**) Effect of RNAi on RGP cell mitotic index, measured as the number of electroporated RGP cells positive for PH3 divided by the total number of electroporated RGP cells. All mitotic figures of RGP cells were located at the ventricular surface, and NDE1 knockdown, as well as NDE1/NDEL1 double knockdown, caused a strong reduction in the mitotic index. Data presented as scatterplot in **c** with bars representing the median±the interquartile range, and as mean±s.e.m. in **d** and **e**. Kolmogorov–Smirnov test for non-parametric distributions used in **c** (*P<0.05, *n*=1,012–1,073 RGP cells). Unpaired *t*-test used in **d** and **e** (*P<0.05, *n*=3 embryonic brains from different mothers). Scale bar, 10 μm.

To test the role of NDE1 compared with NDEL1 in RGP cells, we performed live imaging of brain slice preparations. Control RGPs exhibited normal apical nuclear migration, followed by mitosis at the ventricular surface, and subsequent basal migration (Fig. 2a, Supplementary Movie 1). In marked contrast, NDE1 knockdown severely inhibited INM. Although the RGP cells could initiate apical INM, the nuclei failed to migrate beyond the final 10 μm, never reaching the ventricular surface and entering mitosis (Fig. 2a, Supplementary Movie 2). This behaviour is consistent with a block in late apical nuclear migration during INM, as we have observed with knockdown of Nup133 or CENP-F^25^. NDEL1 knockdown, in contrast, had no discernable effect on INM (Fig. 2a, Supplementary Movie 3). Intriguingly, the double knockdown of both NDE1 and NDEL1 prevented the majority of RGP nuclei from initiating apical INM (Fig. 2a; Supplementary Movie 4). By live imaging, there was no evidence for increased apoptotic events among any of the knockdowns (Supplementary Movies 1–4).

Consistent with these results, fixed tissue analysis revealed that NDE1 knockdown caused a significant accumulation of RGP soma at 10–15 μm from the ventricle (Fig. 2b–d). However, NDE1/NDEL1 double knockdown caused an accumulation of RGP soma further than 30 μm from the ventricle (Fig. 2b–d). Although there is a small but significant difference in the RGP somal distributions between control and NDEL1 knockdown (Fig. 2c), this is not reflected when the groups of somal distances are examined at prescribed distances (Fig. 2d).

Our results, together, indicate that knockdown of either NDE1 or NDEL1 inhibits postmitotic neuronal migration. NDE1 knockdown—but not NDEL1 knockdown—blocks the latter stage of apical INM. Nonetheless, combined NDE1 and NDEL1 knockdown arrests RGP nuclei much further away from the ventricular surface than is observed for NDE1 knockdown alone, suggesting an unexpected synergistic function of the knockdown at a potentially earlier stage of the cell cycle.

### Cell cycle effects of NDE1 and NDEL1 RNAi

To address this issue, and to determine how changes to INM progression may affect RGP cell proliferation and neurogenesis, we examined the effects of NDE1 and NDEL1 RNAi on cell cycle progression. By preventing nuclear migration to the ventricular surface of the developing brain, we predicted that NDE1 knockdown, but not NDEL1 knockdown, would prevent RGP cells from dividing, identifying a potential NDE1-specific microcephaly mechanism. To test for effects on mitosis, we stained sections from electroporated brains for phosphohistone-H3 (PH3) to mark mitotic cells. We observed a severe reduction in the mitotic index in both the NDE1 knockdown and NDE1/NDEL1 double knockdown conditions (Fig. 2e), but no effect from NDEL1 knockdown alone (Fig. 2e). We note that all mitotic events in control electroporated RGPs occurred at the ventricular surface. Importantly, we observed no examples of mitotic divisions of RGP cells away from the ventricular surface under any of the knockdown conditions.

**Figure 3:**
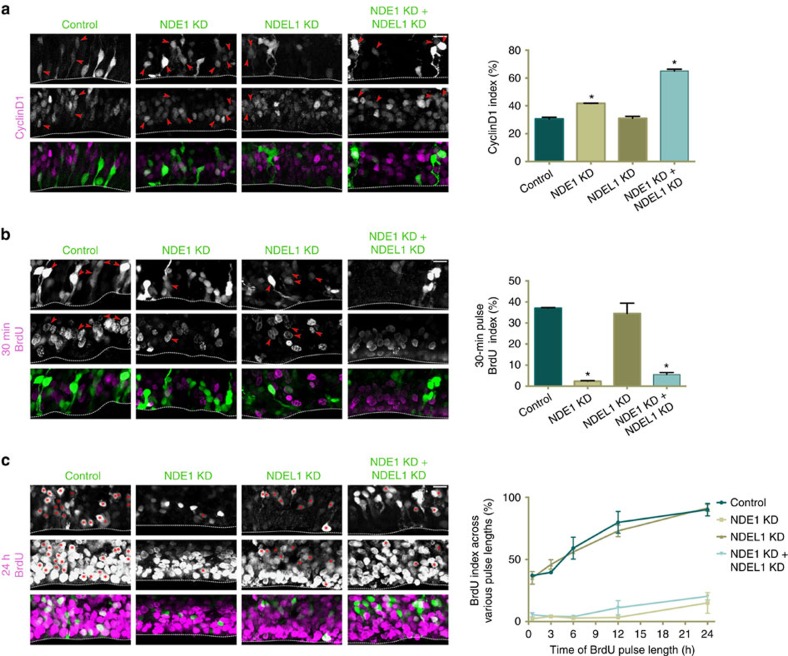
Double knockdown of NDE1/NDEL1 arrests cells at the G1-to-S transition. E16 rat embryonic brains were electroporated with shRNAs for the various conditions and examined at E19. CyclinD1 staining was used to mark G1 cells, a 30 min pulse of BrdU used to mark S-phase cells, and BrdU pulses of varying length were used to distinguish RGPs that are arrested during the cell cycle from those actively cycling. The CyclinD1 and BrdU indices used in quantification were calculated as the amount of electroporated radial glia progenitors (RGPs) positive for either marker divided by the total number of electroporated RGP cells. (**a**) NDE1 knockdown caused a small but significant increase in CyclinD1-positive RGP cells, and the NDE1/NDEL1 double knockdown caused an even more substantial doubling of CyclinD1-positive RGP cells. There was no apparent difference between NDEL1 knockdown and control conditions. CylinD1-positive RGP cells tended to have soma located further away from the ventricular surface. Arrowheads mark electroporated RGP nuclei positive for CyclinD1. (**b**) NDE1 and NDE1/NDEL1 double knockdown caused a reciprocal and severe decrease in BrdU-labelled RGP cells. Again there was no significant difference between NDEL1 knockdown and control conditions. Arrowheads mark electroporated RGP nuclei positive for BrdU. (**c**) BrdU pulses of varying length revealed an increase in BrdU incorporation among control RGPs and NDEL1 knockdown RGPs, in contrast to the very minimal increase in BrdU incorporation over 24 h in the NDE1 and NDE1/NDEL1 knockdown conditions. Unpaired *t*-tests comparing knockdown conditions at each hour revealed no significant difference between control and NDEL1 knockdown, or NDE1 and NDE1/NDEL1 knockdown, while those two pairings were significantly different at every time point observed. Asterisks mark electroporated RGP cells positive for BrdU. Data are presented as mean±s.e.m. Unpaired *t*-tests used to compare conditions, *P<0.05, *n*=3 embryonic brains from different mothers. Scale bar, 10 μm in **a**–**c**. See Supplementary Figs 2 and 3 for further information regarding proliferation status cell cycle of knockdown RGP cells. Dashed line indicates ventricle surface.

The severe effect of NDE1/NDEL1 double knockdown on the distance of arrested RGP soma from the ventricle (Fig. 2b–d) led us to test whether these cells arrest at an earlier cell cycle stage. Staining for the G1 marker CyclinD1 revealed modest or undetectable increases, respectively, in the number of positive RGP nuclei in the NDE1 or NDEL1 shRNA conditions (Fig. 3a). However, combined NDE1/NDEL1 knockdown doubled the number of CyclinD1-positive cells (Fig. 3a), consistent with a potent G1 accumulation. To test this possibility further, we stained sections for bromodeoxyuridine (BrdU) following a 30 min *in vivo* pulse, which revealed a steep drop in S-phase cells in NDE1 and NDE1/NDEL1 double knockdown conditions (Fig. 3b). Staining for Geminin—a S/G2 marker—indicated a significant increase in G2 cells in the NDE1 knockdown condition, whereas there was a sharp decrease in G2 cells in the NDE1/NDEL1 knockdown, as predicted by CyclinD1 data and live imaging (Supplementary Figure 2A,B). Additional staining for the proliferation marker Ki67 revealed no difference in the fraction of positive RGP cells between conditions (Supplementary Fig. 3A,B). Together, these results indicate a synergistic effect of NDE1/NDEL1 double knockdown on preventing G1-to-S transition, with no evidence of cell cycle exit.

To confirm that these knockdowns are indeed inducing a cell cycle arrest, and not merely a delay in cell cycle progression, we performed a series of BrdU pulses of varying durations. Intraperitoneal injections of BrdU every 3 h, for up to 24 h, revealed that nearly 100% of RGPs electroporated with control vector or NDEL1 knockdown were BrdU positive (Fig. 3c). In striking contrast, we saw only a small increase in the fraction of BrdU incorporating cells over longer pulse lengths in the NDE1 knockdown and NDE1/NDEL1 double knockdown conditions (Fig. 3c). Furthermore, this small increase is likely to result from the necessity of beginning BrdU pulses earlier (only 48 h after electroporation), when the shRNA knockdown is just starting to take effect.

**Figure 4:**
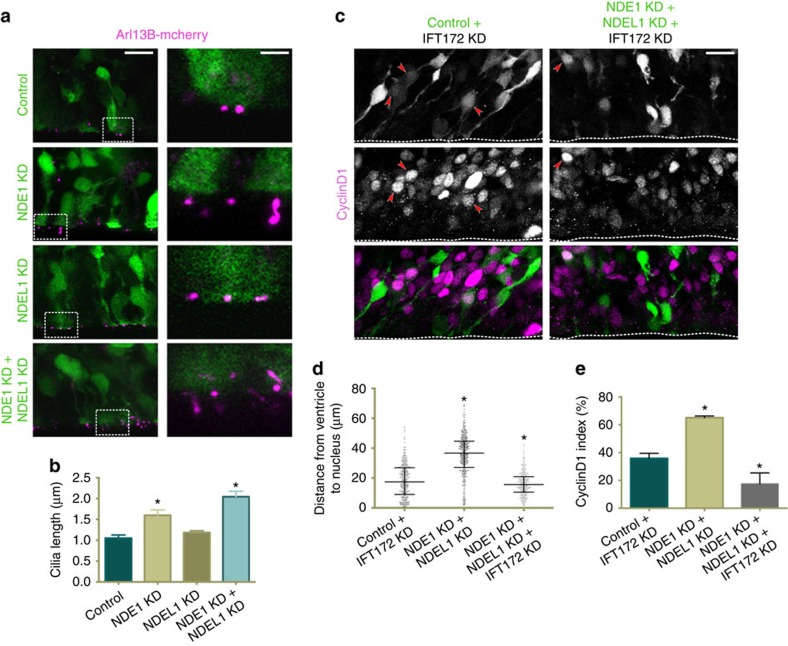
The G1-to-S arrest of radial glia progenitors in the NDE1/NDEL1 double knockdowns disrupts the regulation of primary cilia length. E16 rat embryonic brains were electroporated with the ciliary membrane marker Arl13B and shRNAs to the various conditions described below. All analyses were done at E19. (**a**,**b**) NDE1 knockdown resulted in a significant increase in primary cilia length among electroporated radial glia progenitors (RGPs), though the NDE1/NDEL1 knockdown caused an even greater doubling of primary cilia length. NDEL1 knockdown resulted in no change from control RGP cilia length. (**c**–**e**) Inhibition of primary cilia assembly by knockdown of the intraflagellar transport protein IFT172 rescued the CyclinD1 accumulation seen in the NDE1/NDEL1 double knockdown (**e**). The distribution of apical process lengths of the NDE1/NDEL1/IFT172 triple knockdown more closely mirrored the NDE1 knockdown alone (Fig. 2c). Arrowheads mark electroporated RGP cells positive for CyclinD1. Dashed line indicates ventricle surface. Data are presented as mean±s.e.m. Unpaired *t*-tests used to compare conditions in **b** and **e**, Kolmogorov–Smirnov test used for non-parametric distributions in **d**, *P<0.05, *n*=3 embryonic brains from different mothers. Scale bar, 10 μm in **a** and **c**, and 2.5 μm in insert panels for **a**. Also see Supplementary Figs 4 and 5.

To further understand the mechanism responsible for the G1-to-S arrest seen in the NDE1/NDEL1 double knockdown condition, we began to consider cellular processes necessary for this transition. NDE1 has been reported to be involved in primary cilia resorption in cultured non-neuronal cells, with NDE1 knockdown resulting in ciliary over-elongation^38^. Proper initiation of ciliary resorption was required, in turn, for G0- or G1-to-S-phase progression in proliferating ciliated cells^38^,^39^,^40^. To test whether the G1 accumulation we see in NDE1/NDEL1 double knockdown might reflect a related mechanism, we co-electroporated control and shRNA-encoding plasmids with the ciliary marker Arl13B-mCherry, which reliably labels primary cilia^41^,^42^ (Supplementary Fig. 4A), and examined primary cilia length. NDE1 knockdown caused moderate elongation of RGP cell primary cilia (Fig. 4a,b). In contrast, the combined NDE1/NDEL1 knockdown caused a doubling of primary cilia length compared with controls (Fig. 4a,b). The NDE1/NDEL1 double knockdown also had the greatest effect on increasing primary cilia length in cultured retinal epithelia cells (Supplementary Fig. 5A,B), confirming the synergistic effect of NDE1 and NDEL1 knockdown in this process. To test for a causative role of the primary cilia in the G1-to-S arrest, we knocked down IFT172—a component of the anterograde IFT (intraflagellar transport) complex critical for primary cilia assembly^43^—to inhibit ciliogenesis in RGPs (Supplementary Fig. 4B). Although IFT172 knockdown itself had little effect on the fraction of RGPs in G1, it rescued the G1-to-S block seen in NDE1/NDEL1 double knockdown, as judged by the decrease in the number of CyclinD1 positive RGPs (Fig. 4c,e). Furthermore, analysis of soma position revealed that the majority of RGPs displayed an INM phenotype similar to NDE1 knockdown alone, suggesting that RGPs now progressed through G1 into S-phase and were arrested during apical INM in G2 (Fig. 4c,d). These results further support the necessity of either NDE1 or NDEL1 in initiating resorption of the primary cilia to allow for transition from G1 into S-phase.

### Cross rescue reveals shared and unique functions

**Figure 5:**
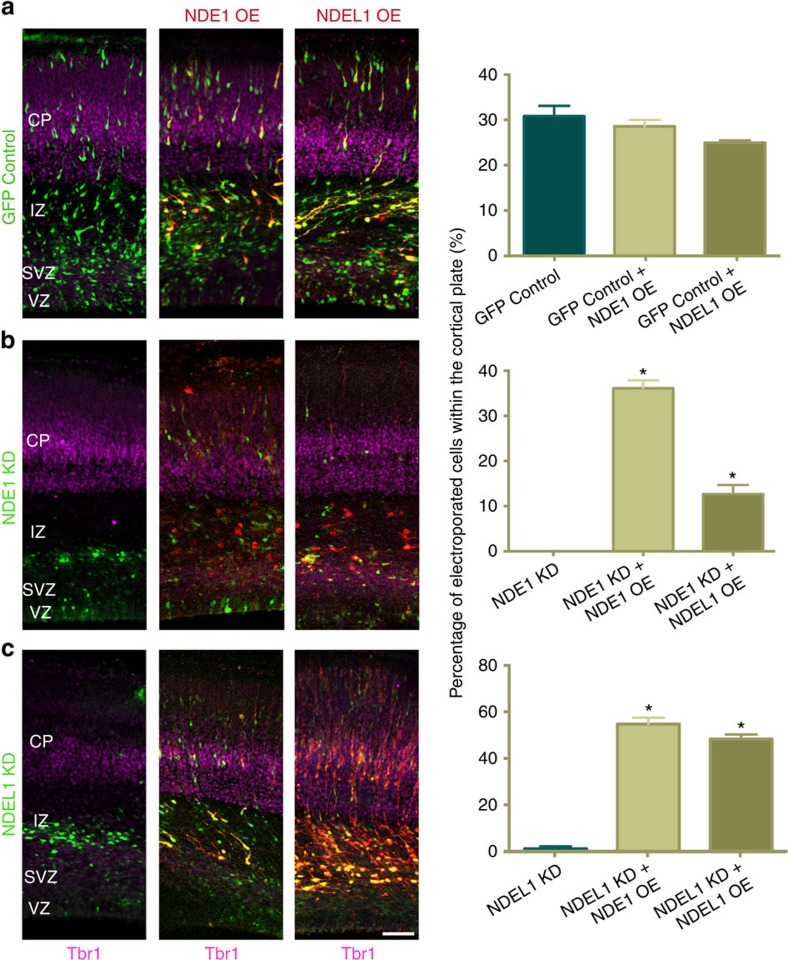
NDE1 or NDEL1 overexpression largely rescues the neuronal migration defects seen after knockdown of either protein. To test for RNAi rescue and functional complementation, embryonic rat brains were co-electroporated at E16 with shRNA to the various conditions with cDNA encoding RNAi-resistant proteins for self-rescue, and standard cDNA for cross-rescue. All the analyses were done at E20. Quantification of the amount of electroporated cells migrating into the cortical plate (CP) is shown at right. (**a**) Overexpression of NDE1 and NDEL1 produced no significant change in the amount of neurons migrating into the CP. (**b**) NDE1 knockdown with overexpression of RNAi-resistant NDE1 rescued neuronal migration into the CP. Overexpression of NDEL1 during NDE1 knockdown partially rescued neuronal migration into the CP. (**c**) Overexpression of RNAi-resistant NDEL1 or NDE1 with NDEL1 knockdown rescued the neuronal migration into the CP, even increasing the fraction of electroporated cells in the CP. Data are presented as mean±s.e.m., and unpaired *t*-tests used for all comparisons, *P<0.05, *n*=3 embryonic brains from different mothers. Scale bar, 50 μm.

**Figure 6:**
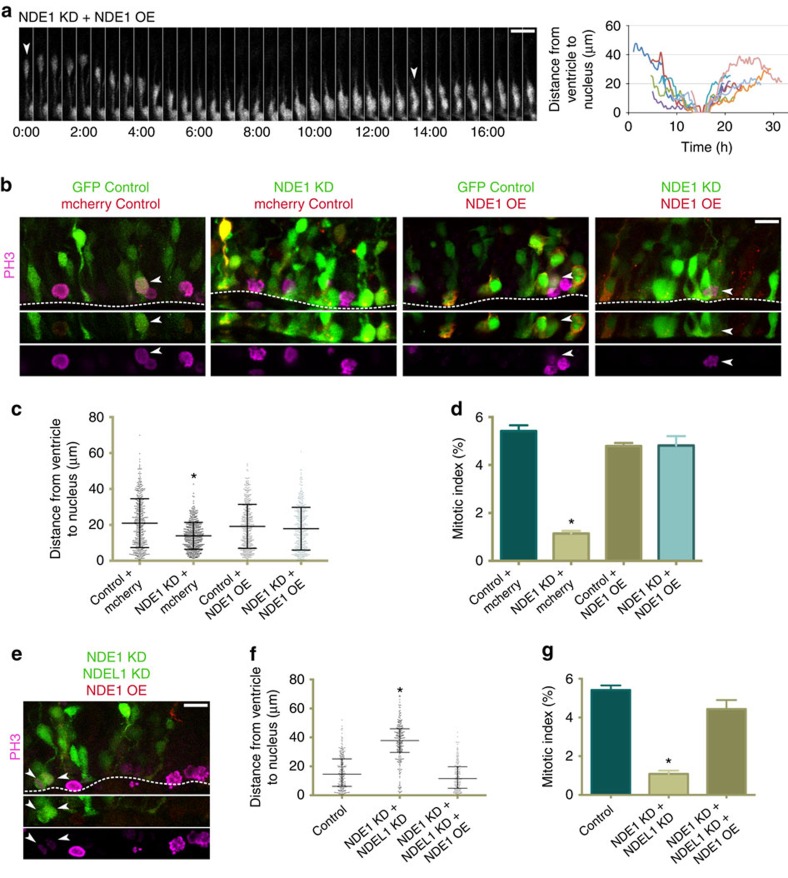
RNAi-resistant NDE1 overexpression rescues all defects seen in radial glia progenitors across knockdown conditions. RNAi-resistant NDE1 was co-electroporated into embryonic rat brains with a GFP control empty vector or along with NDE1 shRNA or NDE1 and NDEL1 shRNAs at E16, and analysed at E20. (**a**) Restoration of apical interkinetic nuclear migration, mitosis and subsequent basal migration of progeny measured by live imaging. Arrowheads marks the radial glia progenitor (RGP) of interest. Montage panels are shown at 30 min intervals. Full movie can be found in Supplementary Material (see Supplementary Movie 5), as well as an additional movie that more clearly displays the two-daughter cell progeny (Supplementary Movie 6). (**b**) Representative images of RGPs stained for PH3 within the VZ in various specified co-expression conditions. Arrowheads mark mitotic electroporated RGPs. Dashed line indicates ventricular surface. (**c**) Soma position of RGPs with RNAi-resistant NDE1 overexpressed during NDE1 knockdown indicates that the somal positioning distribution is rescued. (**d**) Overexpression of RNAi-resistant NDE1 with NDE1 knockdown also rescues the mitotic index. (**e**) Representative image of NDE1/NDEL1 double knockdown with overexpression of RNAi-NDE1, stained for PH3. Arrowheads mark mitotic electroporated RGPs. Dashed line indicates the ventricular surface. (**f**,**g**) Overexpression of RNAi-resistant NDE1 with double NDE1/NDEL1 knockdown rescues the distribution of RGP nuclei in the VZ and restores the mitotic index of RGP cells. Data are presented as scatterplot in **c** and **f** with bars representing the median±the interquartile range, and as mean±s.e.m. in **d** and **g**). Kolmogorov–Smirnov test for non-parametric distributions used in **c** and **f** (*P<0.05, *n*=428–474 RGP cells in **c** and *n*=224–260 RGP cells in **f**). Unpaired *t*-test used in **d** and **g** (*P<0.05, *n*=3 embryonic brains from different mothers). Scale bars, 10 μm. Also see Supplementary Fig. 6.

To define the relative functions of NDE1 and NDEL1 further, in addition to ensuring RNAi specificity, we performed rescue experiments using RNAi-resistant NDE1 and NDEL1 tagged with mCherry (Supplementary Fig. 1C). NDE1 or NDEL1 overexpression each largely rescued the neuronal migration defect seen in knockdown of either NDE1 or NDEL1 (Fig. 5a–c). Although NDEL1 overexpression only partially rescued the neuronal migration defect seen in NDE1 knockdown, these results suggest that each paralogue alone is sufficient for postmitotic migration. Live-imaging of RGP cells further revealed that RNAi-resistant NDE1 fully rescued NDE1 RNAi-inhibited apical INM, mitosis, and subsequent basal migration (Fig. 6a, Supplementary Movies 5 and 6). In addition, fixed imaging results demonstrated full rescue of somal positioning (Fig. 6b,c) as well as mitotic index (Fig. 6d), both in NDE1 RNAi and NDE1/NDEL1 double knockdown cells (Fig. 6e–g; Supplementary Fig. 6). These results indicate that NDE1 alone is sufficient for INM and cell cycle progression in RGPs.

**Figure 7:**
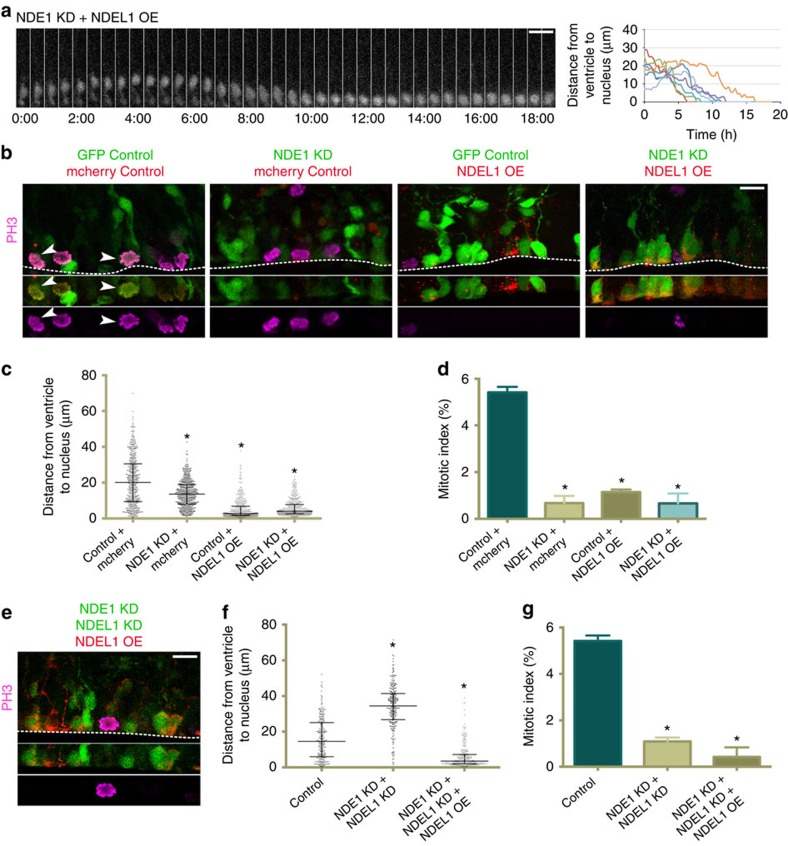
NDEL1 overexpression rescues apical nuclear migration but not mitotic entry in NDE1 deficient radial glia progenitors. cDNA for NDEL1 was co-electroporated into embryonic rat brains with a GFP control empty vector or along with NDE1 shRNA or NDE1 and NDEL1 shRNAs at E16, and analysed at E20. (**a**) NDEL1 overexpression rescues apical interkinetic nuclear migration (INM) in radial glia progenitors (RGPs) where NDE1 has been knocked down, but these cells accumulate at the ventricular surface after apical INM, where they remain for hours without any evidence of mitosis. Montage panels are shown at 30 min intervals. Full movie can be found in Supplementary Movie 7). (**b**) Representative images of NDEL1 overexpression on both a wild-type and NDE1 RNAi background reveals an accumulation of the majority of RGP nuclei at the ventricular surface in a PH3 negative state. Arrowheads mark electroporated cells in mitosis. Dashed line represents the ventricular surface. (**c**) NDEL1 overexpression causes nearly all RGP soma to accumulate at the ventricular surface regardless of whether NDEL1 is overexpressed on a wild-type or NDE1 RNAi background. (**d**) Even though NDEL1 overexpression caused an accumulation of RGP soma at the ventricular surface, the mitotic index remained reduced to a level similar to NDE1 knockdown alone. (**e**) Representative image of RNAi-resistant NDEL1 overexpression with NDE1/NDEL1 double knockdown in RGP cells stained for PH3. Dashed line represents the ventricular surface. (**f**,**g**) RNAi-resistant NDEL1 overexpression with NDE1/NDEL1 double knockdown caused an accumulation of RGP soma at the ventricular surface similar to overexpression of NDEL1 on a wild-type or NDE1-deficient background, and once again failed to rescue the mitotic index. Data are presented as scatterplot in **c** and **f** with bars representing the median±the interquartile range, and as mean±s.e.m. in **d** and **g**. Kolmogorov–Smirnov test for non-parametric distributions used in **c** and **f** (*P<0.05, *n*=405-475 RGP cells in **c** and *n*=245–272 RGP cells in **f**). Unpaired *t*-test used in **d** and **g** (*P<0.05, *n*=3 embryonic brains from different mothers). Scale bars, 10 μm. Also see Supplementary Fig. 7.

We also tested for functional complementation of the two paralogues by overexpressing NDEL1 in NDE1 knockdown RGPs. Live imaging revealed that NDEL1 overexpression fully rescues apical INM, but, surprisingly, the nuclei remained at the ventricle for hours without entering mitosis (Fig. 7a, Supplementary Movie 7). Fixed imaging confirmed the distribution of RGP soma was radically altered by NDEL1 overexpression in NDE1 knockdown RGPs, with nearly all RGP nuclei located adjacent to the ventricle (Fig. 7b,c). Surprisingly, a similar effect on somal distribution was seen with NDEL1 overexpression alone in wild-type rat brain (Fig. 7b,c).

NDE1 and NDEL1 are known to be critical during the progression of mitosis in non-neuronal cells, with inhibition of either paralogue resulting in prometaphase–metaphase arrest^17^,^18^,^19^. Therefore, it was surprising that RGP cells overexpressing NDEL1 alone, or in combination with NDE1 shRNA, were nearly all negative for PH3 (Fig. 7b), with the mitotic index reduced to a level comparable to that resulting from NDE1 knockdown (Fig. 7d). The nuclear envelope in these ventricular surface-arrested cells was also observed to remain intact, as determined by staining for lamin-associated protein 2 (LAP2; Supplementary Fig. 7B). DNA remained uncondensed, as judged by DAPI staining, and centrosomes remained unseparated at the RGP apical endfoot^25^,^44^ (Supplementary Fig. 7A). Similar results were obtained when RNAi-resistant NDEL1 was used to rescue the NDE1/NDEL1 double knockdown (Fig. 7e–g).

Thus, NDEL1 overexpression can compensate for NDE1 during G1-to-S progression and apical INM, but excess NDEL1 induces a premitotic arrest in RGP cells even after the nucleus has reached the ventricular surface, preventing them from entering mitosis.

### BicD2 expression reinforces a novel G2-to-M arrest

Our lab previously found that overexpression of BicD2, the limiting protein for dynein recruitment to the nuclear envelope during early G2 (ref. 25), can rescue RNAi knockdown for genes involved in either the early- or late-G2 dynein recruitment pathways. This resulted in restoration of apical INM as well as subsequent mitosis^25^. Importantly, BicD2 is only targeted to the nuclear envelope during G2, providing an experimental means to restore nuclear envelope dynein recruitment exclusively during this cell cycle stage^31^.

**Figure 8:**
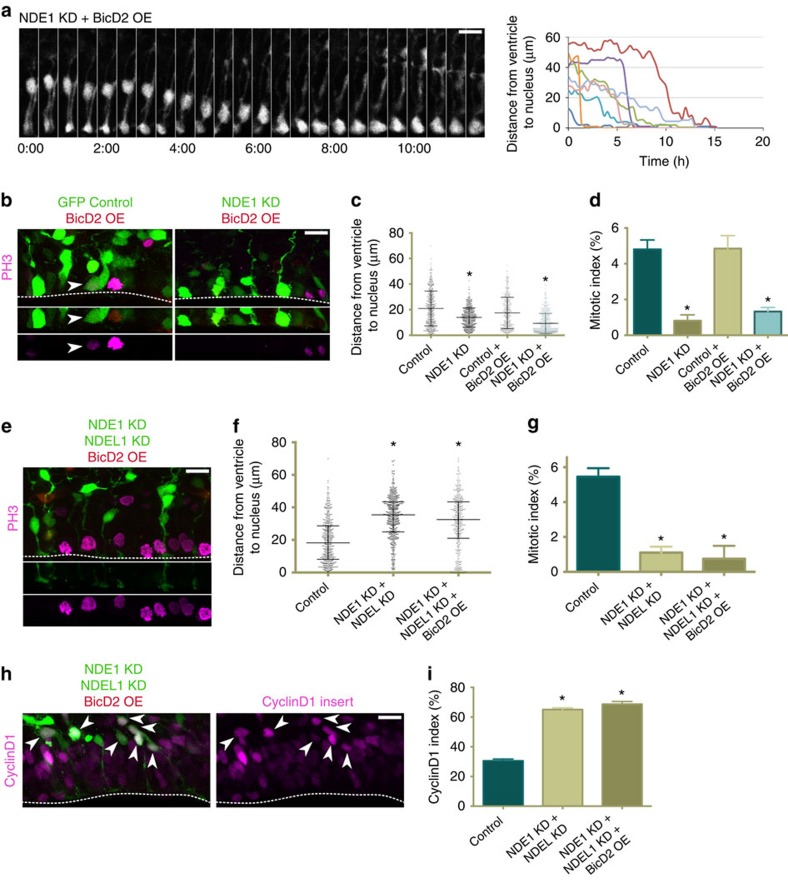
BicD2 overexpression rescues apical nuclear migration but not entry into mitosis in radial glia progenitors depleted of NDE1. cDNA for full-length BicD2 was co-electroporated into embryonic rat brains with a GFP control empty vector or along with NDE1 shRNA or NDE1 and NDEL1 shRNAs at E16, and analysed at E20. (**a**) The overexpression of BicD2 in radial glia progenitors (RGPs) lacking NDE1 restores apical migration, though the soma accumulate at the ventricle for hours without any evidence of mitosis. Montage panels are shown at 30 min intervals. Full movie can be found in Supplementary Movie 8. (**b**) Representative images of BicD2 overexpression on both a wild-type and NDE1 knockdown background with staining for PH3. Arrowheads mark mitoses in electroporated cells. Dashed line indicates ventricular surface. (**c**) BicD2 overexpression did not alter the somal distribution of control RGP cells but caused the vast majority of NDE1 knockdown RGP soma to accumulate at the ventricular surface. (**d**) Despite the accumulation of RGP soma at the ventricle in NDE1 knockdown with BicD2 overexpression, the mitotic index remained reduced. (**e**) Representative image of RGP cells with BicD2 overexpression along with double NDE1/NDEL1 knockdown, stained for PH3. Dashed line indicates ventricle. (**f**,**g**) Overexpression of BicD2 with NDE1/NDEL1 double knockdown fails to rescue the somal distribution pattern or mitotic index of double NDE1/NDEL1 knockdown RGP cells. (**h**,**i**) The same ratio of RGP nuclei were positive for CyclinD1 whether or not BicD2 was overexpressed along with the double NDE1/NDEL1 knockdown, indicating the prominence of the G1-to-S block in the double knockdown, and the G2 specificity of the BicD2 rescue strategy. Arrowheads mark electroporated RGP nuclei positive for CyclinD1. Dashed line indicates ventricle surface. Data are presented as scatterplot in **c** and **f** with bars representing the median±the interquartile range, and as mean±s.e.m. in **d**,**g** and **i**. Kolmogorov–Smirnov test for non-parametric distributions used in **c** and **f** (*P<0.05, *n*=407–591 RGP cells in **c** and *n*=421–487 RGP cells in **f**). Unpaired *t*-test used in **d**,**g** and **i** (*P<0.05, *n*=3 embryonic brains from different mothers). Scale bars, 10 μm. Also see Supplementary Fig. 7.

In the current study, overexpression of BicD2 along with NDE1 shRNA fully rescued apical INM, though, in this case, nuclei accumulated at the ventricle for prolonged periods of time without detectable signs of mitotic entry (Fig. 8a, Supplementary Movie 8). Consistent with these results, the majority of RGP soma in fixed brain sections were located at the ventricular surface (Fig. 8b,c) with intact nuclear envelope, uncondensed DNA and unseparated apical centrosomes (Supplementary Fig. 7A,B). Altogether, these results provide further evidence for the requirement of NDE1 during late-G2 dynein recruitment to the nuclear envelope, and reveal a new NDE1-specific role after completion of apical INM but before mitotic entry. Notably this NDE1 function cannot be rescued by overexpression of NDEL1 or BicD2.

Intriguingly, when both NDE1 and NDEL1 were knocked down and BicD2 overexpressed, the vast majority of RGP nuclei remain arrested far from the ventricular surface in a CyclinD1 positive state (Fig. 8e–i), similar to the double NDE1/NDEL1 knockdown condition without BicD2 overexpression. The mitotic index was again severely reduced (Fig. 8e,g). As BicD2 overexpression acts solely during G2, these results further confirm that most RGP cells subjected to NDE1/NDEL1 double knockdown were indeed arrested during G1 rather than G2, as suggested earlier by the CyclinD1 and primary cilia results.

## Discussion

**Figure 9:**
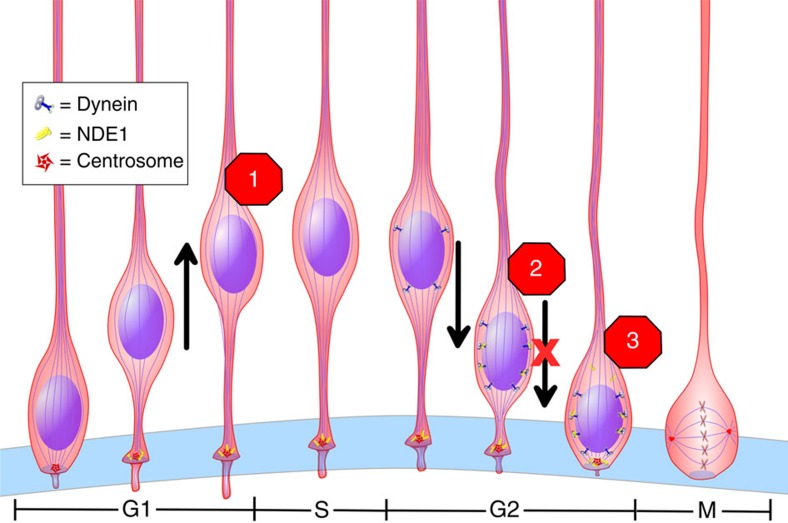
Roles of NDE1 and NDEL1 in the neural progenitor cell cycle. Our data indicate that NDE1 is required for three distinct non-mitotic processes in the radial glia progenitor (RGP) cell cycle: G1-to-S progression (1), apical INM during late G2 (2) and G2-to-M transition (3). NDEL1 overexpression rescues (1) and (2), but not (3). Interference with the RGP cell cycle at any or all of these stages is proposed as an important contributor to microcephaly. Scale of image does not reflect actual duration of cell cycle stages.

NDE1 and NDEL1 are each involved in neocortical development, though genetic analysis in rodents and human patients have revealed distinct phenotypic and disease causing potential for the two paralogues^2^,^6^,^7^,^45^. Direct analysis of the functional similarities and differences between the two genes remains very limited, making an understanding of the factors leading to these developmental abnormalities challenging. Although NDE1, in particular, has been implicated in a severe form of human microcephaly^2^,^7^,^8^,^9^, the specific underlying mechanism is uncertain. We find here that NDE1 and NDEL1 can substitute for each other in diverse aspects of neurogenesis and neuronal migration. Our data, however, reveal a strongly predominant role for NDE1 in RGP cell behaviour. We identify three distinct non-mitotic stages of RGP cell cycle progression that are susceptible to reduction in NDE1 or both NDE1/NDEL1, each of which should lead to a marked decrease in neurogenesis (Fig. 9). These observations should help resolve the mechanisms responsible for *NDE1*-associated microcephaly and other forms of the disease.

We find that NDE1 RNAi causes substantial numbers of RGP nuclei to become arrested during apical INM. This effect blocks the RGP soma from reaching the ventricular surface, preventing these cells from ever entering mitosis^25^. The distance from the ventricular surface at which the NDE1 knockdown nuclei arrest is similar to that seen for CENP-F and Nup133 knockdown, the two upstream factors in the late-G2 pathway for recruitment of cytoplasmic dynein to the nuclear envelope^25^,^31^,^34^. Though definitive G2-immunohistochemical markers are lacking, our live imaging results strongly support G2 arrest, and a failure to enter mitosis at the ventricular surface. Analysis of a *NDE1* null mouse, however, revealed ectopic mitotic figures^6^, which we do not see in either NDE1, NDEL1, or combined knockdown RGP cells. Conceivably, the residual mitoses in the *NDE1* null mouse could be associated with intermediate progenitor cells located within the SVZ, or result from compensatory upregulation of *NDEL1* in the *NDE1* null mouse.

In examining functional redundancy between NDE1 and NDEL1, we uncovered another premitotic NDE1-specific role during cell cycle progression. According to our data, NDE1 is required to act following completion of apical INM to avoid a block in G2-to-M transition. This arrest occurs before histone-H3 phosphorylation, nuclear envelope breakdown, DNA condensation, centrosome separation and centrosome release from the apical endfoot. Importantly, NDEL1 was unable to compensate for NDE1 knockdown at this stage. In fact, overexpression of NDEL1 induced a similar phenotype, suggesting a dominant-negative effect, perhaps involving formation of heterodimers with NDE1 (ref. 46). By utilizing BicD2 overexpression, we were also able to force NDE1 knockdown RGP cells to complete INM, yet these cells remained unable to enter mitosis. This stands in contrast to BicD2 rescue of CENP-F knockdown in RGP cells, which were able to complete apical INM and enter mitosis^25^, and suggests an as-yet unidentified, spatially sequestered apical signal responsible for RGP mitotic entry. There has been one prior report of a NDE1 mutation leading to a partial increase in the G2 population of non-neuronal cells^2^. How these observations may relate to ours is uncertain. Nonetheless, our data point to a NDE1-specific role in the G2-to-M transition, separate from its role in the late-G2 pathway of recruiting dynein to the nuclear envelope and identify an underlying difference between the two paralogues.

Primary cilia have been implicated in cell cycle progression in a number of cell lines and developing tissue^38^,^40^,^47^,^48^. Even though the primary cilia membrane has been shown to be retained for most of the RGP cell cycle^42^, previous reports have provided evidence that resorption of primary cilia needs to be initiated for ciliated cells to transition into S-phase^38^,^40^. NDE1, in particular, has been implicated in this process in ciliated non-neuronal cell lines and during development of the zebrafish head^38^,^49^. A similar block in the G1-to-S transition in RGP cells was observed in our experiments, accompanied by the over-elongation of primary cilia. An additionally intriguing aspect of our observations was that this arrest was exacerbated by the double knockdown of NDE1/NDEL1, unlike apical INM inhibition, which was readily apparent when only NDE1 was knocked down. This enhanced G1/S block with double knockdown suggests that each paralogue alone can contribute to initiating ciliary resorption in RGPs, and rescue of this phenotype by inhibiting ciliogenesis indicates that dysregulation at the primary cilia is causal of the G1/S block in the NDE1/NDEL1 double knockdown in RGP cells.

The precise function of primary cilia in radial glia remains unresolved. Knockout mice for KIF3A or IFT88, proteins integral for cilia formation, have largely normal brain development^50^,^51^, and we detected no difference in CyclinD1 labelling or somal distribution in our IFT172 knockdown conditions alone. Nonetheless, lack of primary cilia is very different from dysregulation of the ciliary growth cycle and cilia over-elongation, which may explain why development occurs largely normally without primary cilia, while aberrations in primary cilia length/resorption may block cell proliferation. This view is further supported by a report of a block to RGP cell cycle progression caused by knockdown of TcTex1, which also affects ciliary resorption^40^. Nevertheless, it remains to be resolved whether NDE1 and NDEL1 are playing a role in recruiting specific regulators of the primary cilia, whether they facilitate retrograde intraflagellar transport, or whether they play an entirely separate role in primary cilia regulation/signalling.

Microcephaly arises from impaired proliferation of the progenitor cells, resulting either from defects in S-phase, or more commonly, in mitotic progression^1^. The current study, however, reveals three alternative mechanisms associated with a severe microcephaly-causing gene, *NDE*1, and is the first to link aberrations in apical INM to human disorders of corticogenesis. Relative to rodents, development of the human neocortex relies on an expanded outer subventricular zone containing many additional RGPs^52^,^53^,^54^. All of these, however, must derive from apical RGPs in the ventricular zone that must undergo INM to successfully proliferate. Both NDE1 and NDEL1 are known to serve critical functions during mitosis, in particular during spindle alignment^17^ and chromosome segregation^16^,^18^,^19^. Although NDE1 is likely required for these functions in RGPs, our data show that acute NDE1 knockdown arrests RGPs before mitotic entry. Our data, therefore, indicate that the inability to reach mitosis is an equally or even more important cause of microcephaly than an arrest within mitosis itself.

Patients with *NDE1* mutations exhibit altered cortical lamination, in addition to reduced brain size^2^,^7^,^8^,^9^, which raises the question of whether defects in proliferation are solely accountable for the laminar deficits seen by magnetic resonance imaging. In addition to the three major cell cycle roles for NDE1 identified in RGP proliferation, NDE1 and NDEL1 were each shown to be crucial for the migration of the postmitotic neuronal precursors into the cortical plate. Thus, these changes in postmitotic migration likely explain the subsidiary cortical laminar dysplasia seen in many of the patients with *NDE1* microcephaly^2^,^7^.

Altogether, it appears that the two dynein regulatory proteins NDE1 and NDEL1 have both overlapping and distinct functions during mammalian neocortical development. Though differences in protein expression and posttranslational modification likely contribute to differences as well, our results imply that there is at least one role for NDE1, at the G2-to-M transition, for which NDEL1 cannot functionally compensate. Further work will be required to define the nature of this very interesting function.

## Methods

### *In utero* electroporation

Plasmids were transfected by intraventricular injection into the cranium of rats at embryonic day 16 (E16) and electroporated. In brief, an E16 gravid rat was anaesthetized via intraperitoneal injection of ketamine (75–95 mg kg^−1^) and xylazine (5 mg kg^−1^), and placed on a temporary heat source. Pain management was provided by the administration of buprenorphine (0.05 mg kg^−1^) and bupivicaine (2 mg kg^−1^). Laparotomy was performed and the uterine horns transilluminated with a fibre-optic light source to identify the embryonic cerebral ventricles. DNA was mixed with a coloured non-toxic dye for easy visualization and 1 μl of DNA constructs (1–2 μg μl^−1^) was injected into the ventricular space using a high gauge needle made from a glass capillary tube. Post injection, five pulses of electrical current (50 V, 5 ms each, with 1 s intervals) were applied by placing electrodes on the external wall of the uterine horn such that the positive electrode is angled along the lateral aspect of the neocortex adjacent to the lateral ventricle targeted by the injection. The uterus was returned to the abdominal cavity, the incision site closed by suture of the abdominal rectus muscle and closure of the skin via abdominal wound clips. The rats were monitored every day post surgery until the desired developmental time point, and buprenorphine (0.05 mg kg^−1^) was provided every 8–12 h for the first 48 h of recovery for pain management^55^. All animal protocols were approved by the Institutional Animal Use and Care Committee at Columbia University.

### Cortical slice preparation and immunostaining

Either 3 or 4 days following the *in utero* electroporation procedures (see figure legends for precise dates of each experiment), the gravid rat was anaesthetized again using ketamine (75-95 mg kg^−1^) and xylazine (5 mg kg^−1^). Upon proper anaesthetic induction, a laparotomy at the site of the previous incision was performed and the uterus exposed. The embryos were excised from the uterus, the brains dissected out and placed overnight in 4% paraformaldehyde (PFA) fixative dissolved in PBS at 4 °C. Following fixation, the brains were embedded in 4% agarose in PBS and sliced by a vibratome (Zeiss) in 80 μm thick coronal sections. The sections were incubated in blocking solution containing PBS and 0.3% Triton X-100 supplemented with 3% normal donkey serum for 1 h. Primary antibodies were incubated overnight in blocking solution at 4 °C, sections were washed 3 × in PBS and secondary antibodies in blocking solution were incubated for 2 h at room temperature. The sections were mounted on slides using Aqua-Poly/Mount (Polysciences, Inc). For BrdU immunostaining, BrdU (Sigma-Aldrich) was injected intraperitoneally (20 mg ml^−1^) into the gravid mother once every 3 h for cumulative pulses of either 3, 6, 12 or 24 h duration. For the 30 min BrdU pulse, the brains were sliced according to the live imaging protocol (described below), immersed in BrdU containing media (20 μg ml^−1^) for 30 min before fixation with PFA. For BrdU immunohistochemistry, brain slices were first incubated in 2 N HCl for 25 min at 37 °C. The slices were then washed in 0.1 M sodium borate for 10 min and then PBS, before antibody incubation.

### Live imaging

The dissected rat brains were embedded in 4% low melting agarose diluted in artificial cerebrospinal fluid^55^ and were sliced into 300 μm coronal sections as described above. The slices were placed on 0.4 μm, 30 mm diameter Millicell-CM inserts (Millipore) in cortical culture medium containing 25% Hanks balanced salt solution, 47% basal MEM, 25% normal horse serum, 1 × penicillin/streptomycin/glutamine (GIBCO BRL), and 30% glucose. The slice was transferred to a 50 mm glass-bottom dish and imaged on an IX80 laser scanning confocal microscope (Olympus FV100 Spectral Confocal System) at intervals of 10 min for up to 24 h.

### RNAi and DNA constructs

shRNA constructs were designed to target internal gene sequences uniquely against NDE1 or NDEL1 and contained in a pRetro-U6G vector (Cellogenetics, MD, USA), which co-expressed GFP and the shRNA target sequences. The target sequence for NDE1 is 5′- GCGTTTGAATCAAGCCATTGA -3′. The target sequence for NDEL1 is 5′- GATGGTGAAGATATACCGGAT -3′. Both target sequences were conserved in mice and rat. Two target sequences for IFT172 were used in combination: 5′- GCGGCCATCAACCACTATATT -3′ and 5′- GCTGCTGATCTCTCATTACTA -3′ (Sigma). Empty vectors of pEGFP-C1 and pmCherry-N1 were used as controls (Clontech).

For overexpression of NDE1 and NDEL1, mouse cDNA to either protein was fused to a mCherry fluorescent reporter (mCherry-C1-NDE1 and mCherry-N1-NDEL1) and subcloned into a pCAGEN vector driven by CAG promoter (provided by Connie Cepko (Addgene plasmid #11160)) using XhoI and NotI restriction sites. Five silent point mutations were made in the cDNA for both NDE1 and NDEL1 so that each was RNAi resistant for their respective shRNAs using KOD Hot Start (Millipore). shRNA sequences directed at the opposite paralogue had at least six incongruent nucleotides in the target sequence and, therefore, were specific to either protein. pIRES-dsRed-BicD2 was described in Hu *et al*., 2013. pCAG-Arl13B-mCherry was provided by Dr Kathryn Anderson.

The short interfering (siRNA) smart pools (GE-Dharmacon) used for NDE1 and NDEL1 RNAi in the human retinal epithelial cell line (hTERT–RPE1–GFP–CSAP, a gift from Dr. Iain Cheeseman) were as follows: siGENOME NDE1 (54820) siRNA—5′- GGACCCAGCUCAAGUUUAA -3′; 5′- GGAAAGAUCUGGCGAUGAC -3′; 5′- GGAGGAAGAAGCUAACUAU -3′; 5′- GGAGGGAAGCCGAGAAUAU -3′; and siGENOME NDEL1 (81565) siRNA—5′- GAAGCUAGAGCAUCAAUAU -3′; 5′- GCUAGGAUAUCAGCACUAA -3′; 5′- GGACCAAGCAUCACGAAAA -3′; 5′- GCACAAAGUUCUCUCGAUC -3′.

### Cell analyses and western blotting

Rat C6 brain glioma cells were cultured in DMEM supplemented with 10% FBS, 1% penicillin/streptomycin and maintained at 37 °C with 5% CO_2_. Transfection with shRNAs against NDE1 and NDEL1 was performed using a Lonza nucleofector kit V and an Amaxa Nucelofector according to the manufacturer's instructions. The cells were collected 72 h later and sorted using fluorescence-activated cell sorting (FACS) to isolate GFP-positive cells. The collected cells were lysed on ice in RIPA buffer containing DTT and a protease inhibitor cocktail (Sigma). Purified cell lysates were loaded on a polyacrylamide gel and transferred to a polyvinylidene difluoride membrane. The membrane was blocked in PBS+0.5% powdered milk, incubated with primary antibodies to NDE1/NDEL1 (Abnova; 1:1000) and α-tubulin (Sigma; 1:2000) diluted in blocking solution+0.05% Tween for 2 h, washed and incubated with secondary LI-COR antibodies.

hTERT-immortalized retinal pigment epithelial cell line expressing the centriolar and ciliary axoneme marker GFP–CSAP (hTERT-RPE1–GFP–CSAP; a gift from Dr. Iain Cheeseman) were grown in DMEM/F12 supplemented with 10% FBS, 1% penicillin/streptomycin and maintained at 37 °C with 5% CO2. Transfections with siRNA smart pools were performed using Lipofectamine RNAiMAX (Life Technologies) according to the manufacturer's instructions. Forty-eight hours following transfection, the cells were washed in PBS re-transfected with the siRNA pools and incubated with serum-free media for an additional 48 h to promote ciliogenesis. The cells were then washed in PBS, fixed in 4% PFA for 8 min and permeabilized with 0.15% Triton X-100 in PBS for 2 min. After blocking in donkey serum for 30 min, the cells were incubated with primary antibodies followed by incubation with fluorophore-conjugated secondary antibodies.

### RNA isolation and quantitative RT–PCR analysis

C6 cells were transfected with NDE1/NDEL1 shRNA plasmids and rescue constructs using an Amaxa Nucleofactor as described for western blot. Seventy-two hours following transfection, GFP fluorescent cells were sorted using a FACSAria Cell Sorter (BD). Positive cell lyses, mRNA to cDNA synthesis and quantitative PCR were performed using the Power SYBR Green Cells-to-Ct Kit (Ambion/Thermofisher). cDNA were analysed using quantitative PCR using an ABI 7900 HT quantitative PCR machine. Primers were designed to have TMs of about 60 degrees and to generate amplicons of 70 to 200 base pairs, separated by at least one intron. Three replicas were done for each condition per experiment and the experiments were performed in triplicates. Target cDNA levels were analysed by the comparative cycle (Ct) method and values were normalized against β-actin and Gapdh expression levels. The primers used in this study were: β-actin FW: 5′- CCCGCGAGTACAACCTTCT -3′; β-actin RV: 5′- CGTCATCCATGGCGAACT -3′; Gapdh FW: 5′- CAACTCCCTCAAGATTGTCAGCAA -3′; Gapdh RV: 5′- GGCATGGACTGTGGTCATGA -3′; NDE1 FW: 5′- GAAAGAGCCAAACGAGCCAC -3′; NDE1 RV: 5′- TCAGTCTCTGCACAGATTCTA -3′ * (1 mismatch with mouse sequence); NDEL1 FW: 5′- GAAGTGGAGGCGTTAAAGGA -3′; NDEL1 RV: 5′- CCAGTGAAACTATTGTTGCCC -3′.

### Antibodies

Antibodies used in this study were mouse monoclonal against phosphohistone H3 (Abcam, ab14955, 1:500 dilution), phospho-vimentin (Millipore, 05-774, 1:250 dilution), Ki67 (Millipore, MAB4190, 1:250 dilution), centrin3 (Abnova, H00001070-M01, 1:250 dilution), glutamylated tubulin (Addipogen, GT335, 1:250 dilution), rabbit polyclonal against CyclinD1 (Thermo Scientific, RM-9104, 1:250 dilution), Geminin (Santa Cruz, sc-13015, batch J2615, 1:250 dilution), Lamin-associated protein 2 (Santa Cruz, sc-28541, 1:500 dilution), Tbr1 (Abcam, ab31940, 1:500 dilution), Arl13B (Proteintech, 17711-1-AP, 1:250 dilution), and rat monoclonal against BrdU (Abcam, ab6326, 1:250 dilution). Donkey fluorophore-conjugated secondary antibodies (Jackson Labs, 1:500 dilution) were used together with DAPI (4',6-diamidino-2-phenylindole, Thermo Scientific, 62248, 1:1,000 dilution). NDE1 (Abnova, H00054820-M01, 1:1,000 dilution) and α-tubulin (Sigma; 1:2,000) were used for immunoblotting. To develop in a LICOR system, fluorescent secondary antibodies were acquired from Invitrogen (dilution 1:10,000) and Rockland (dilution 1:10,000) to use for western blotting.

### Imaging and statistical analysis

All images were collected with an IX80 laser scanning confocal microscope (Olympus FV100 Spectral Confocal System). Brain sections were imaged using a × 60 1.42 N.A. oil objective or a × 10 0.40 N.A. air objective. All drawings were composed using Inkscape open source software. All images were analysed using ImageJ software (NIH, Bethesda, MD, USA). Distance and primary cilia length measurements were also performed using this software. Live-imaging movies were constructed on ImageJ at 12 f.p.s., with each frame representing a 10 min progression in real time. Tracings were made by measuring the distance from the ventricular surface to the bottom of the soma—the same as was used for fixed imaging analysis—every 20 min, corresponding to every two frames in the movies. All statistical analysis was performed using Prism (GraphPad Software, La Jolla, CA, USA). For fixed analysis of distances of RGP soma from the ventricle (apical process length), distributions were represented as scatterplots with bars plotting the median and interquartile range. This was done to provide a more comprehensive representation of the data, since all conditions failed the D'Agostino & Pearson omnibus normalcy test. Due to the nonparametric nature of these distributions, Kolmogorov–Smirnov tests were used for all comparisons of experimental conditions to the corresponding control condition. The ROUT method was used to identify any outliers, none of which were found across the conditions. Measurements of the distance between RGP nuclei and ventricular surface were made from three different embryonic rat brains, each from a different mother. For mitotic index, CyclinD1 ratio, BrdU ratio, Ki67 ratio and the fraction of electroporated cells reaching the cortical plate, all data are plotted as the mean with standard error of mean. Measurements were all made from at least three different embryonic rat brains across at least two separate operations per condition. Animals from all successful operations were included in the analysis. For the index measurements, at least 100 RGP cells were counted from at least three slices per brain. For results with Gaussian distributions, comparisons were made first using analysis of variance tests to determine whether there was a significant difference, followed by unpaired *t*-tests to compare individual experimental conditions to the appropriate control condition. For all non-Gaussian distributions, a Kruskal–Wallis test was used first, followed by Kolmogorov–Smirnov tests or Dunn's multiple comparison tests for direct comparison of individual conditions. For all analyses, significance was accepted at the level of *P*<0.05.

### Data availability

The authors declare that all relevant data supporting the findings of this study are either provided in the Article and Supplementary files or available from the authors on request.

## Additional information

**How to cite this article:** Doobin, D. J. *et al*. Severe *NDE1*-mediated microcephaly results from neural progenitor cell cycle arrests at multiple specific stages. *Nat. Commun.* 7:12551 doi: 10.1038/ncomms12551 (2016).

## Supplementary Material

Supplementary Information Supplementary Figures 1-8

Supplementary Movie 1 E16 rat embryonic brain electroporated with a GFP control plasmid were continuously imaged at E19 for 16hrs at 10 min intervals. Scale bar, 15μm. Arrowheads indicate mitoses.

Supplementary Movie 2 E16 rat embryonic brain electroporated with NDE1 RNAi were continuously imaged at E19 later for 17hrs, 10min at 10 min intervals. Scale bar, 10μm.

Supplementary Movie 3 E16 rat embryonic brain electroporated with NDEL1 RNAi were continuously imaged at E19 for 18hrs, 30min at 10 min intervals. Scale bar, 20μm. Arrowheads indicate mitoses.

Supplementary Movie 4 E16 rat embryonic brain electroporated with NDE1 and NDEL1 RNAi and continuously imaged at E19 for 16hrs, 50min at 10 min intervals. Scale bar, 20μm

Supplementary Movie 5 E16 rat embryonic brain electroporated with NDE1 RNAi and RNAi-resistant NDE1 were continuously imaged at E20 for 16hrs 30 min at 10 min intervals. Arrowhead marks the RGP nucleus where apical INM is clearly rescued, though mitotic division occurs within the z-plane and is difficult to see. Scale bar, 15μm.

Supplementary Movie 6 E16 rat embryonic brain electroporated with NDE1 RNAi and RNAi-resistant NDE1 were continuously imaged at E20 for 15hrs, 50min at 10 min intervals. Arrowhead marks the RGP nucleus where apical INM is rescued, and the second arrow represents the second daughter RGP following mitosis. Scale bar, 15μm.

Supplementary Movie 7 E16 rat embryonic brain electroporated with NDE1 RNAi and overexpressing NDEL1 were continuously imaged at E20 for 19hrs, 10min at 10 min intervals. Arrowhead marks the RGP nucleus where apical INM is rescued, and the arrow represents the apical endfoot. Scale bar, 10μm.

Supplementary Movie 8 E16 rat embryonic brain electroporated with NDE1 RNAi and overexpressing NDEL1 were continuously imaged at E20 for 19hrs, 10min at 10 min intervals. Arrowhead marks the RGP nucleus where apical INM is clearly rescued, and the arrow represents the apical endfoot. Scale bar, 10μm.
