# Supplementary material for: Severe NDE1-mediated microcephaly results from neural progenitor cell cycle arrests at multiple specific stages
Source: Nat Commun. 2016 Aug 24;7:12551. doi: 10.1038/ncomms12551 (PMC4999518; doi:10.1038/ncomms12551)
Supplement: Supplementary Information — Supplementary Figures 1-8 [file ncomms12551-s1.pdf]

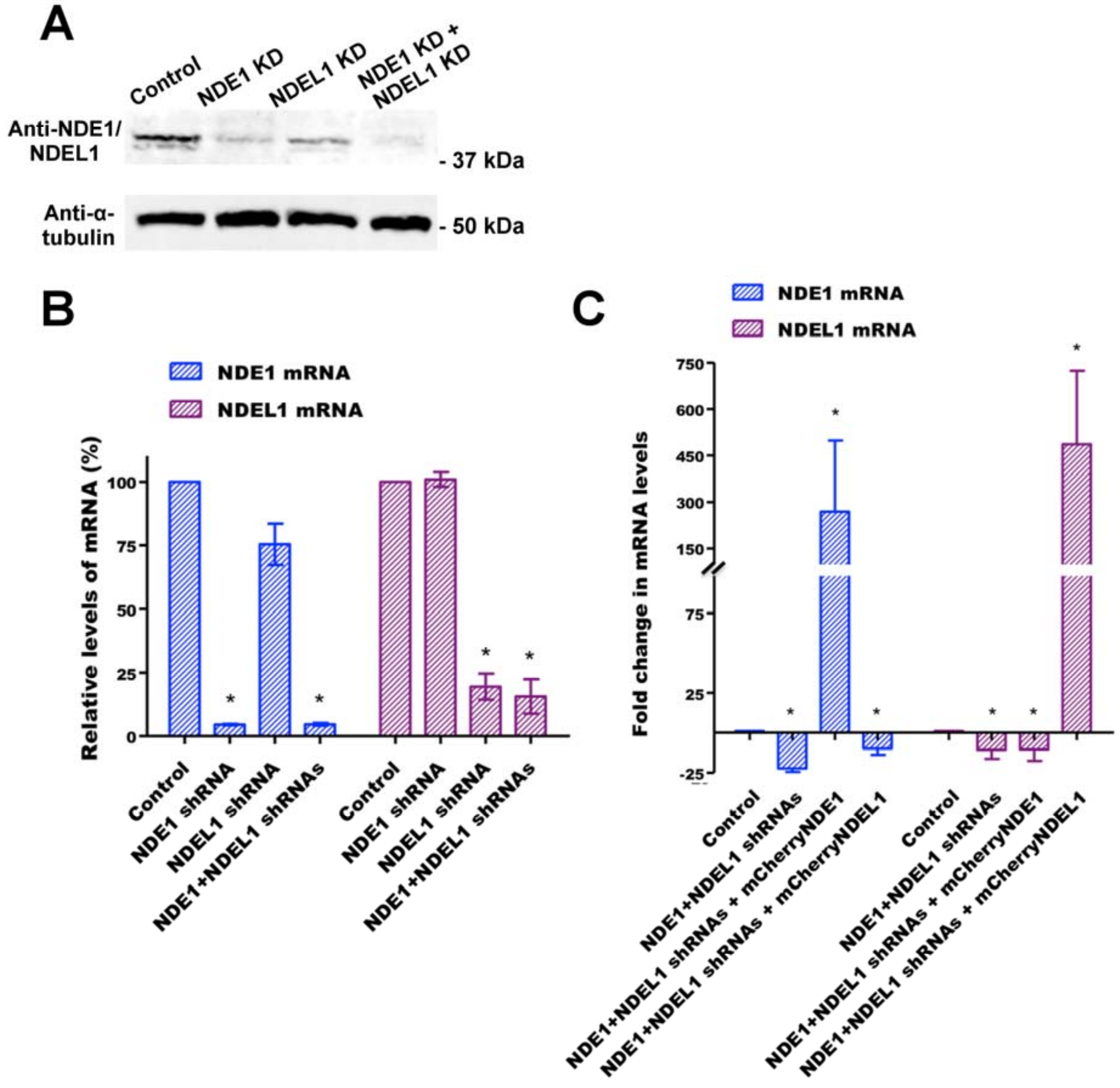

Supplementary Figure 1, related to Figure 1: Confirmation of shRNA knockdown efficiency

(A) Lysate from C6 rat glioma cells transfected with control or shRNA constructs 72 hours prior to lysing confirms successful knockdown of NDE1 and NDEL1 by western blot.

(B) mRNA levels from C6 cell lysate were used to measure NDE1 and NDEL1 shRNA efficiency. NDE1 shRNA reduced the levels of NDE1 mRNA to <5% of the original, with some minimal effects on NDEL1 mRNA levels. NDEL1 shRNA reduced the levels of NDEL1 mRNA to <20% of the original.

(C) Rescue of NDE1 and NDEL1 mRNA levels by RNAi-insensitive cDNA of either NDE1 or NDEL1.

Data presented as mean ± SEM. Statistical analyses performed using Kruskall-Wallis with Dunn's multiple comparison test. * for p<0.05

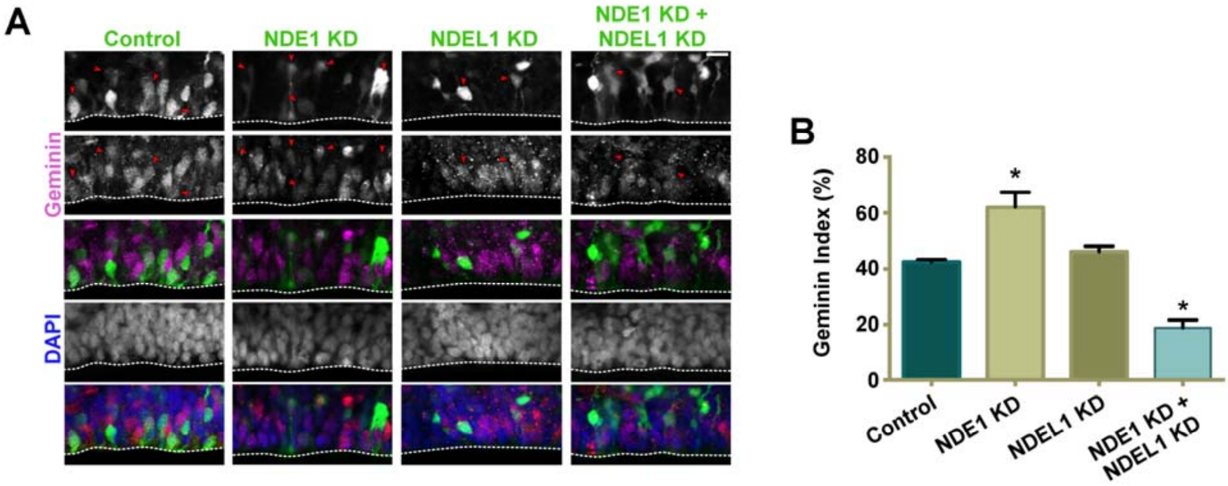

Supplementary Figure 2, related to Figure 3: The effects of NDE1 and NDEL1 knockdowns on RGPs in G2

E16 rat embryonic brains were electroporated with shRNAs to the various conditions described below. All analysis was done at E19.

(A) Representative images of RGP cells across the various specified conditions stained for the S/G2 marker Geminin.

(B) Quantification of the Geminin index across conditions, defined at the ratio of electroporated RGP cells positive for Geminin divided by the total number of electroporated RGP cells. There was a significant increase in Geminin labeling between the NDE1 KD and control conditions, and a significant decrease between the NDE1/NDEL1 KD and control conditions. Data are presented as mean ± SEM. Unpaired t-tests used to compare conditions, * for p<0.05, n=3 embryonic brains from different mothers. Scale bar, 10μm in (A).

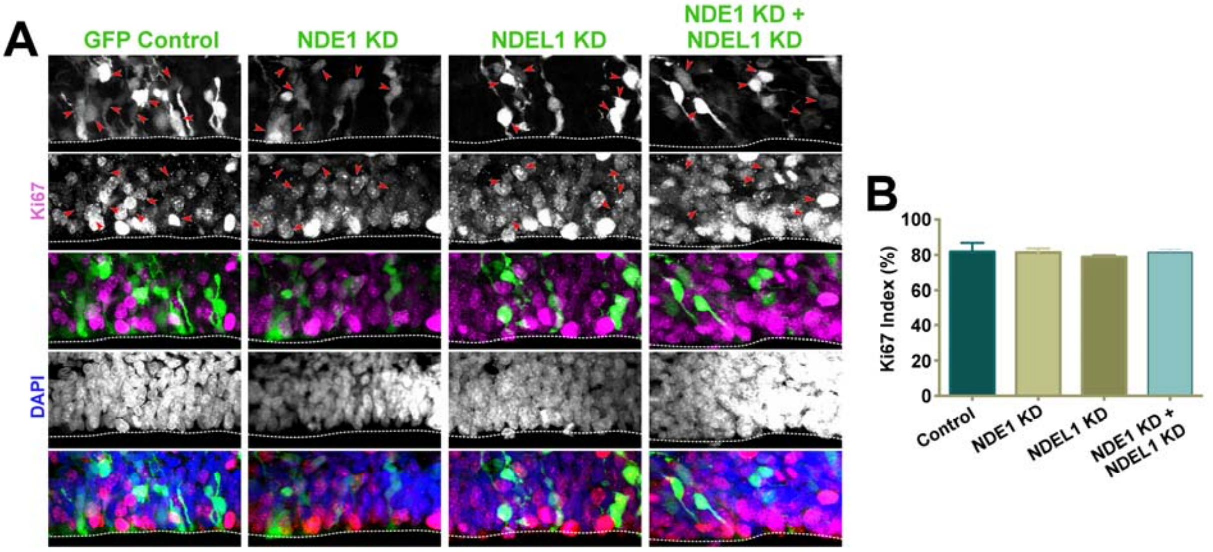

Supplementary Figure 3, related to Figure 3: The effects of NDE1 and NDEL1 knockdowns on actively proliferating RGPs

E16 rat embryonic brains were electroporated with shRNAs to the various conditions described below. All analysis was done at E19.

(A) Representative images of RGP cells across the various specified conditions stained for the proliferation marker Ki67.

(B) Quantification of the Ki67 index across conditions, defined as the ratio of electroporated RGP cells positive for Ki67 divided by the total number of electroporated RGP cells. No significant differences were found between conditions.

For all panels, RGP cells were identified on the basis of morphology and the possession of apical process. Arrowheads mark double labeled RGP cells. Scale bar, 10µm. 2-channel composite is presented with electroporation signal in green and immunocytochemical signal in magenta. 3-channel composite is presented with electroporation signal in green, immunocytochemical signal in red, and DAPI in blue. Data presented as mean ± SEM. Unpaired *t*-test used for comparisons, * for $p<0.05$, $n=3$ embryonic brains from different mothers. Data are presented as mean ± SEM. Unpaired *t*-tests used to compare conditions, $n=3$ embryonic brains from different mothers. Scale bar, 10µm in (A).

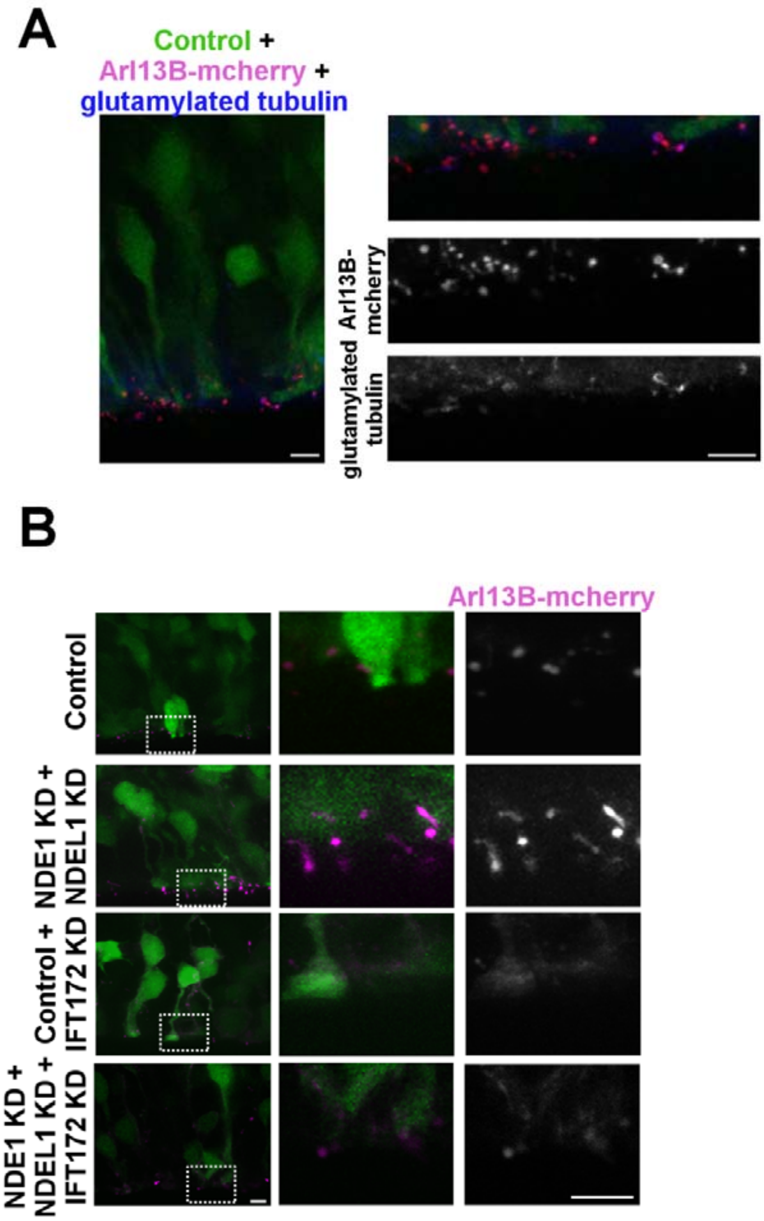

Supplementary Figure 4, related to Figure 4: IFT172 knockdown overcomes the deregulation of primary cilia length seen upon NDE1/NDEL1 double knockdown

E16 rat embryonic brains were electroporated with the ciliary membrane marker Arl13B and shRNAs to the various conditions described below. All analysis was done at E19.

(A) Control staining of brain slices expressing GFP empty vector and Arl13B-mCherry (magenta) with glutamylated tubulin - a ciliary axoneme and basal body marker.

(B) Inhibition of primary cilia assembly by knockdown of the intraflagellar transport protein IFT172, decreases the occurrence of elongated primary cilia in the NDE1/NDEL1 double knockdown. Scale bars represent 5µm.

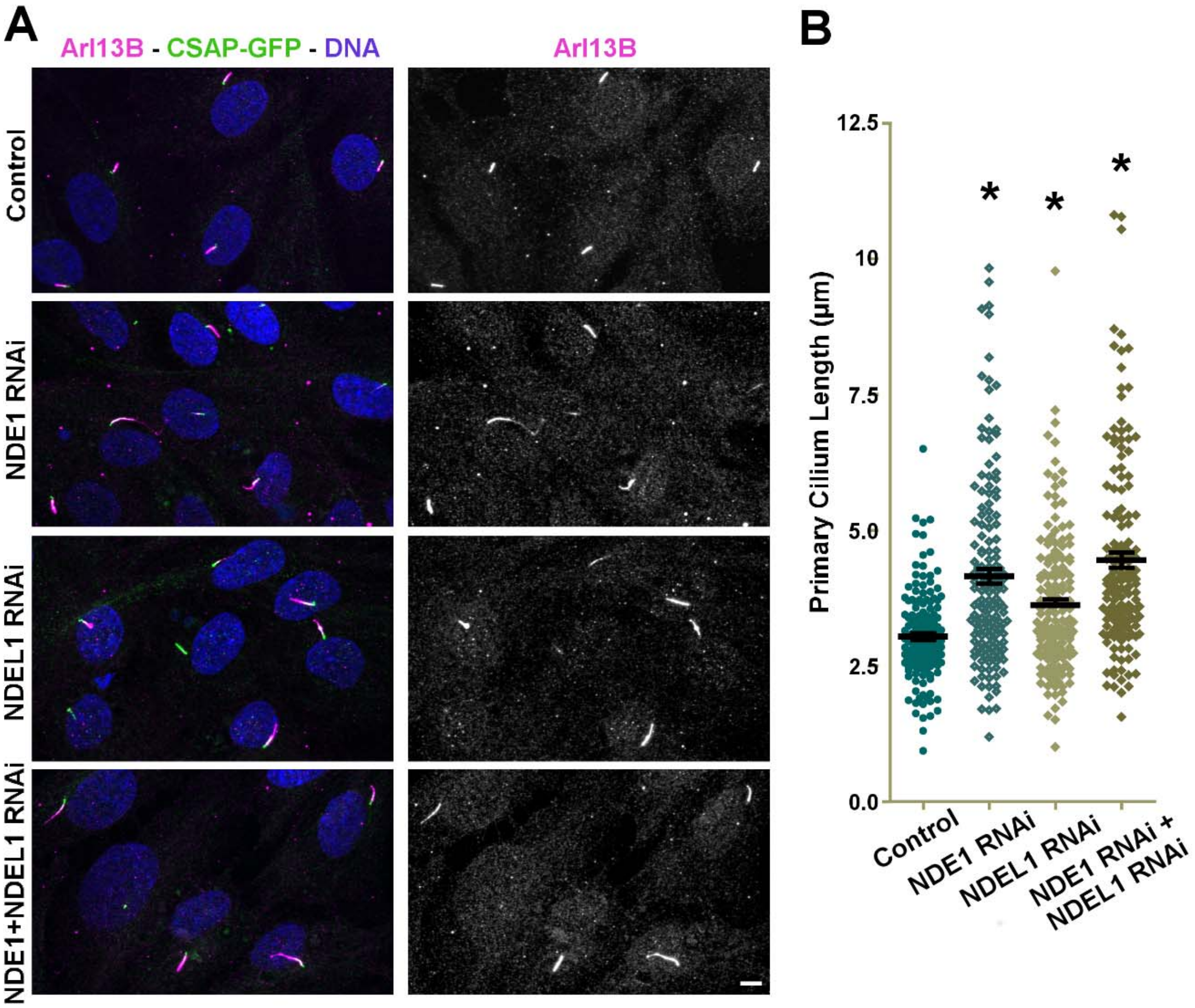

Supplementary Figure 5, related to Figure 4: NDE1, NDEL1, or knockdown of both proteins leads to increased primary cilia length in human cells.

(A) Immunofluorescence micrographs of serum-starved control and RNAi treated hTERT-RPE1-GFP-CSAP cells stained as labeled. Scale bar, 5µm.

(B) Primary cilia length in control and RNAi conditions as labeled. RPE1 cells (n=3x50). Error bars represent ± SEM. Statistical analyses performed using Kruskall-Wallis with Dunn's multiple comparison test, $p<0.05$, values labeled with *.

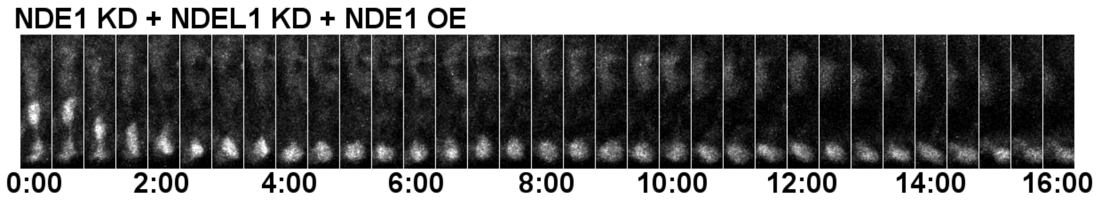

Supplementary Figure 6, related to Figure 6: NDE1 overexpression restores apical INM and mitotic capabilities to RGPs with NDE1/NDEL1 double knockdown

Live-imaging example of an RGP cell exposed to NDE1 and NDEL1 shRNA, as well as overexpressed, RNAi-insensitive NDE1, undergoing apical interkinetic nuclear migration (INM) and mitosis. Cytokinesis is visible in the panel at hour 15.

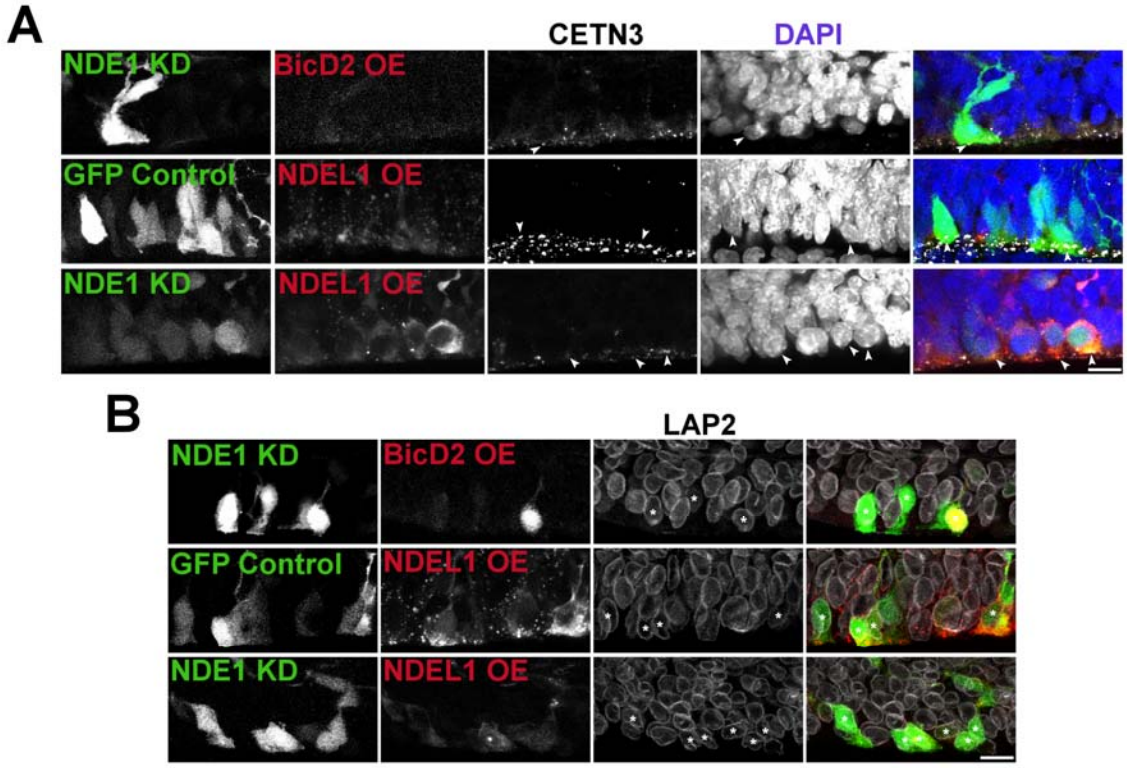

Supplementary Figure 7, related to Figures 7 and 8: RGP cells with nuclei that have accumulated at the ventricular surface appear to be truly premitotic.

(A) Staining for centrin3 (CETN3) and DAPI revealed that RGP nuclei that had accumulated at the ventricular surface had uncondensed chromosomes and centrosomes that remained un-separated and localized at the ventricular surface. Arrowheads mark centrosomes and DAPI signal of electroporated RGP cells.

(B) Staining for lamin-associated protein 2 (LAP2) across the specified conditions reveals that these RGP nuclei had intact nuclear evidence with no evidence of invagination or breakdown. Asterisks mark the nuclei of electroporated RGP cells. Scale bar, 10µm.

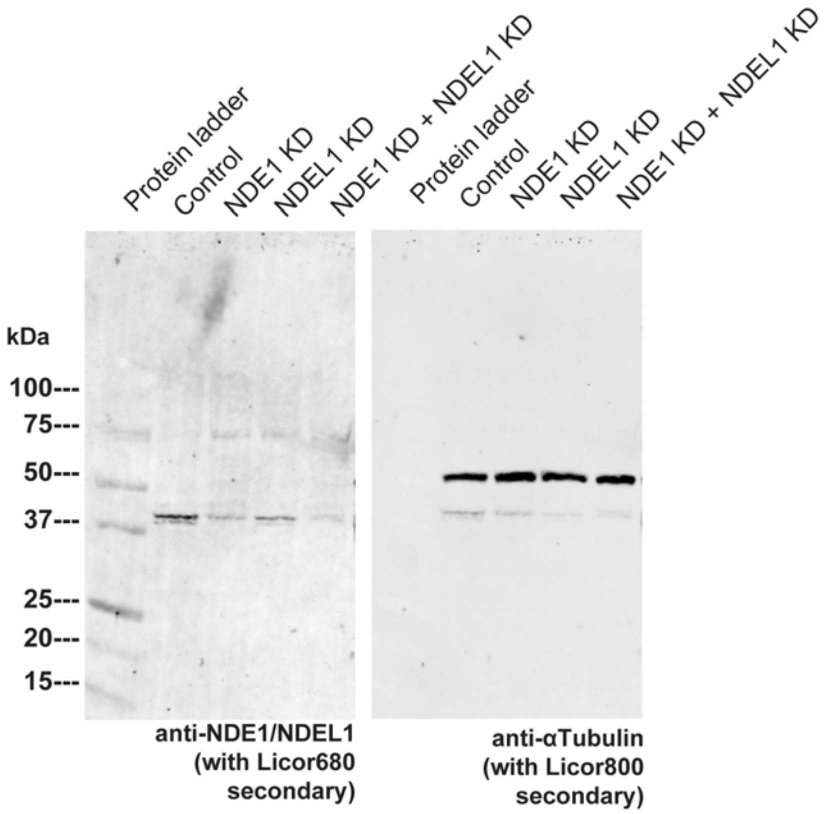

Supplementary Figure 8, related to Supplementary Figure 1: Uncropped western blot of anti-NDE1/NDEL1 and anti-α-Tubulin.
